# Supplementary figures and images for: Association of urban inequality and income segregation with COVID-19 mortality in Brazil
Source: PLoS One. 2022 Nov 15;17(11):e0277441. doi: 10.1371/journal.pone.0277441 (PMC9665357; doi:10.1371/journal.pone.0277441)

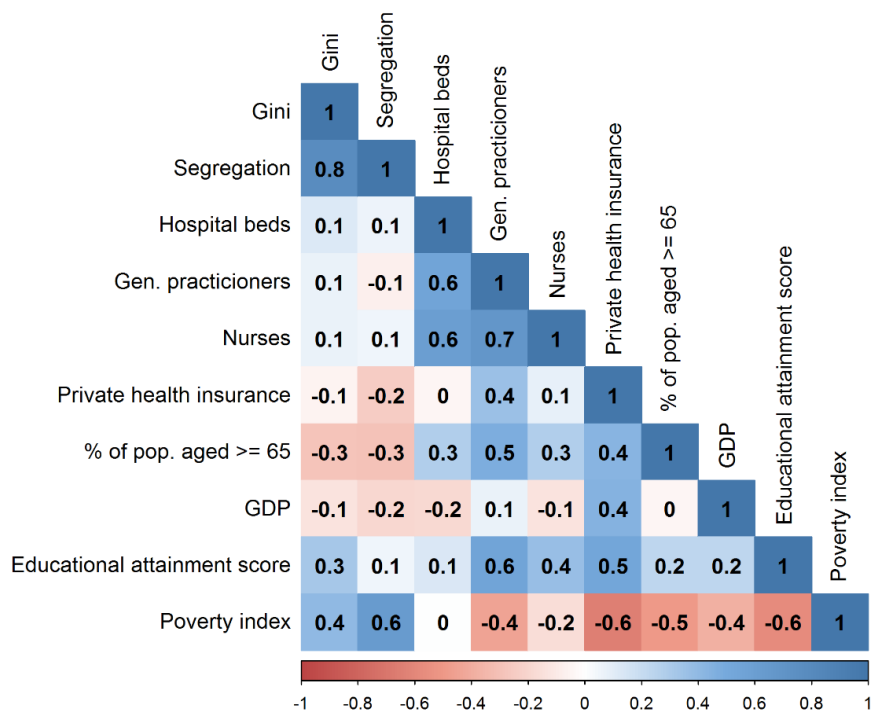

**S1 Fig. Correlation matrix between all of the exposures and covariates used in the paper.**

Supplement: S1 Fig — (PDF) [file pone.0277441.s002.pdf]

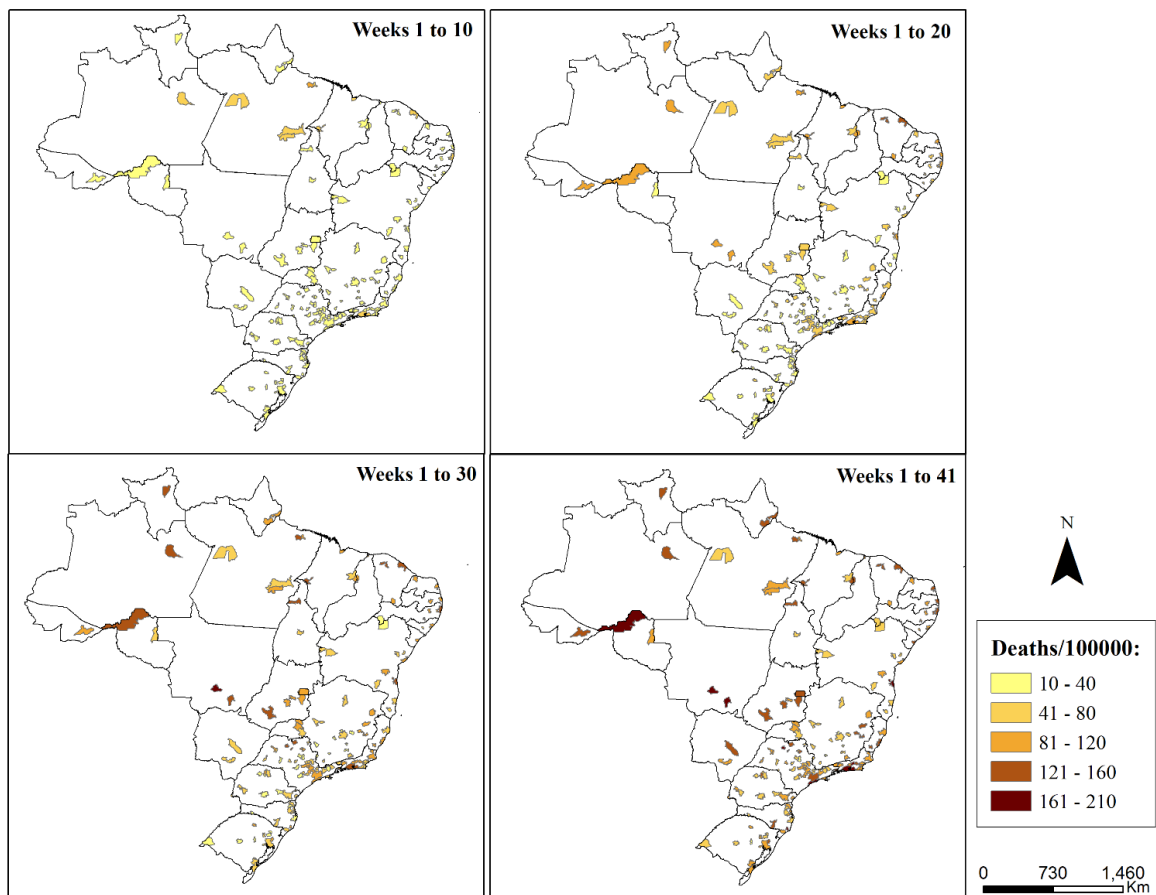

**S3 Fig. City-level COVID–19 mortality rates, aggregated across weeks.**

Supplement: S3 Fig — (PDF) [file pone.0277441.s004.pdf]
