# Supplementary material for: Association of urban inequality and income segregation with COVID-19 mortality in Brazil
Source: PLoS One. 2022 Nov 15;17(11):e0277441. doi: 10.1371/journal.pone.0277441 (PMC9665357; doi:10.1371/journal.pone.0277441)
Supplement: S2 Fig — *DAG built using DAGitty®. (PDF) [file pone.0277441.s003.pdf]

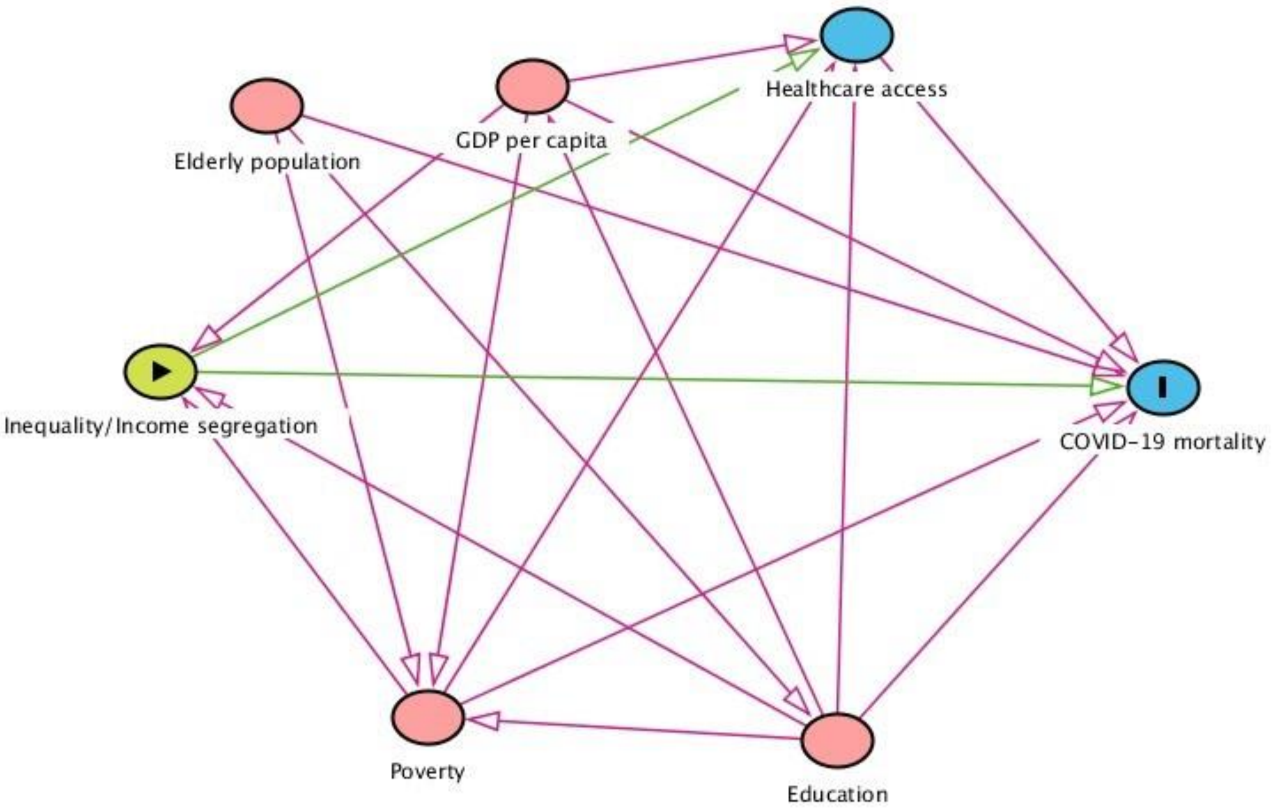

S1 Fig. Direct acyclic graph (DAG)* of the causal associations and relationships between income inequality and income segregation with COVID-19 mortality.

*DAG built using DAGitty®.
