# Supplementary material for: Association of urban inequality and income segregation with COVID-19 mortality in Brazil
Source: PLoS One. 2022 Nov 15;17(11):e0277441. doi: 10.1371/journal.pone.0277441 (PMC9665357; doi:10.1371/journal.pone.0277441)
Supplement: S4 Fig — (PDF) [file pone.0277441.s005.pdf]

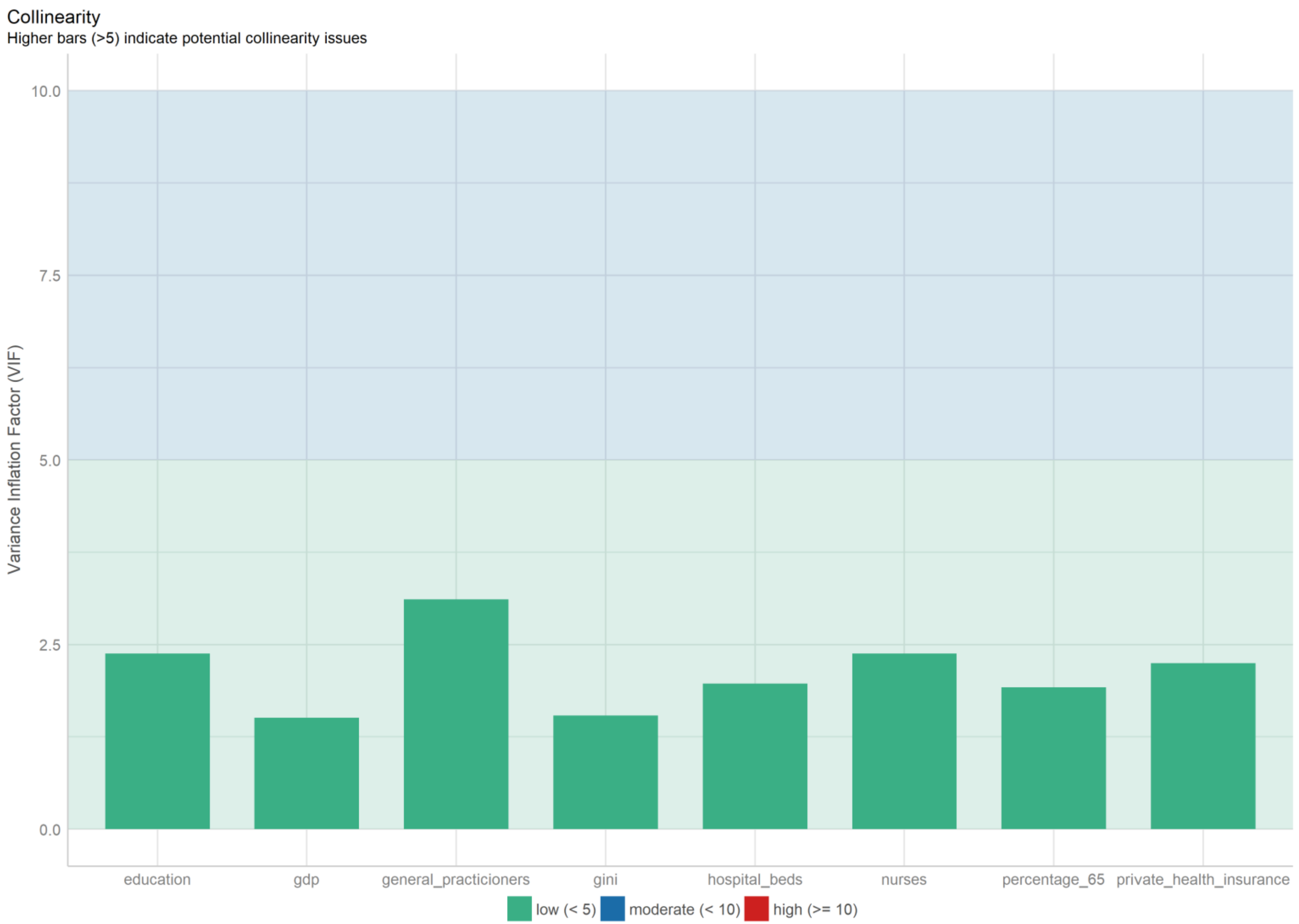

S1 Fig. Variance Inflation Factors of the exposure and covariates for the fully adjusted negative binomial model estimating the association between Gini and COVID-19 mortality.
