# Supplementary material for: Association of urban inequality and income segregation with COVID-19 mortality in Brazil
Source: PLoS One. 2022 Nov 15;17(11):e0277441. doi: 10.1371/journal.pone.0277441 (PMC9665357; doi:10.1371/journal.pone.0277441)
Supplement: S6 Fig — Dots are point estimates (rate ratios), and confidence bars are 95% confidence intervals. Model 1 = unadjusted model, Model 2 = adjusted by social environment covariates, Model 3 = fully adjusted model (social environment covariates and healthcare indicators). (PDF) [file pone.0277441.s007.pdf]

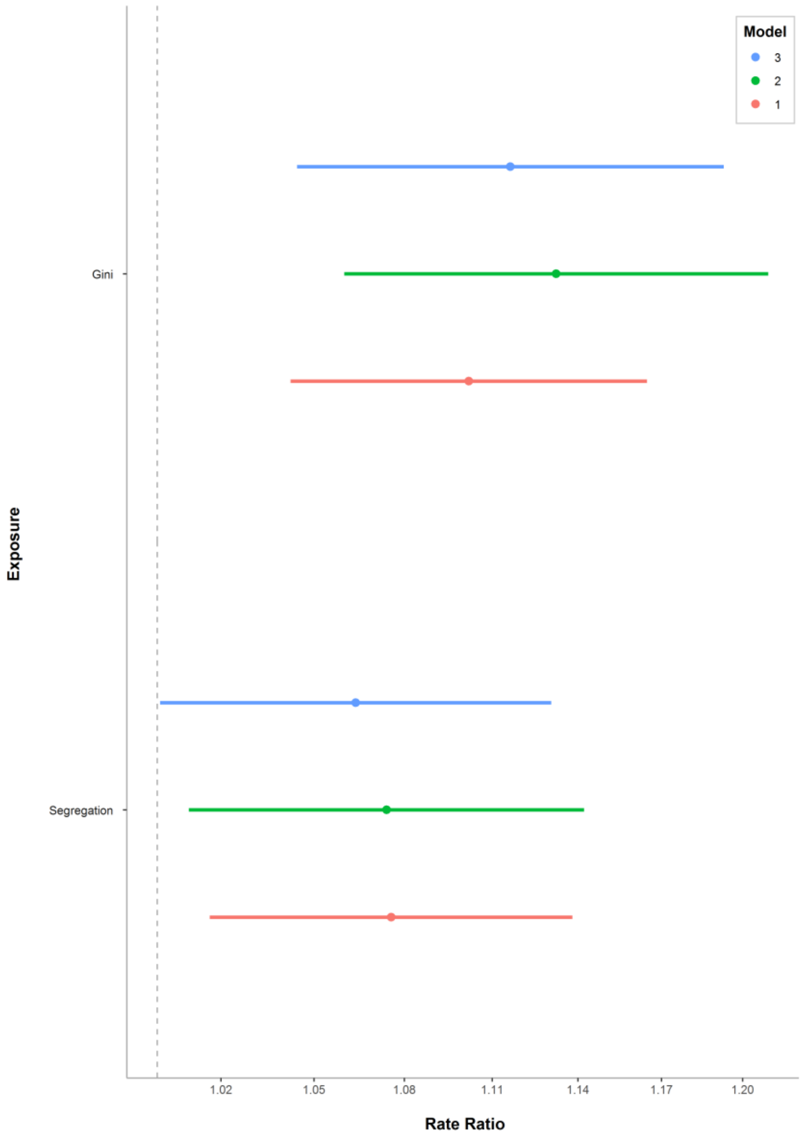

S6 Fig. Forest plot of estimated associations between COVID-19 mortality rates and income inequality and segregation for no-balancing sample.

Dots are point estimates (rate ratios), and confidence bars are 95% confidence intervals. Model 1 = unadjusted model, Model 2 = adjusted by social environment covariates, Model 3 = fully adjusted model (social environment covariates and healthcare indicators).
