# Supplementary material for: Association of urban inequality and income segregation with COVID-19 mortality in Brazil
Source: PLoS One. 2022 Nov 15;17(11):e0277441. doi: 10.1371/journal.pone.0277441 (PMC9665357; doi:10.1371/journal.pone.0277441)
Supplement: S7 Fig — The solid black line represents the LOESS fit, with its 95% confidence interval given in the shaded bands. CO = Centro-Oeste (Central-West), N = Norte (North), NE = Nordeste (Northeast), S = Sul (South), SE = Sudeste (Southeast). (PDF) [file pone.0277441.s008.pdf]

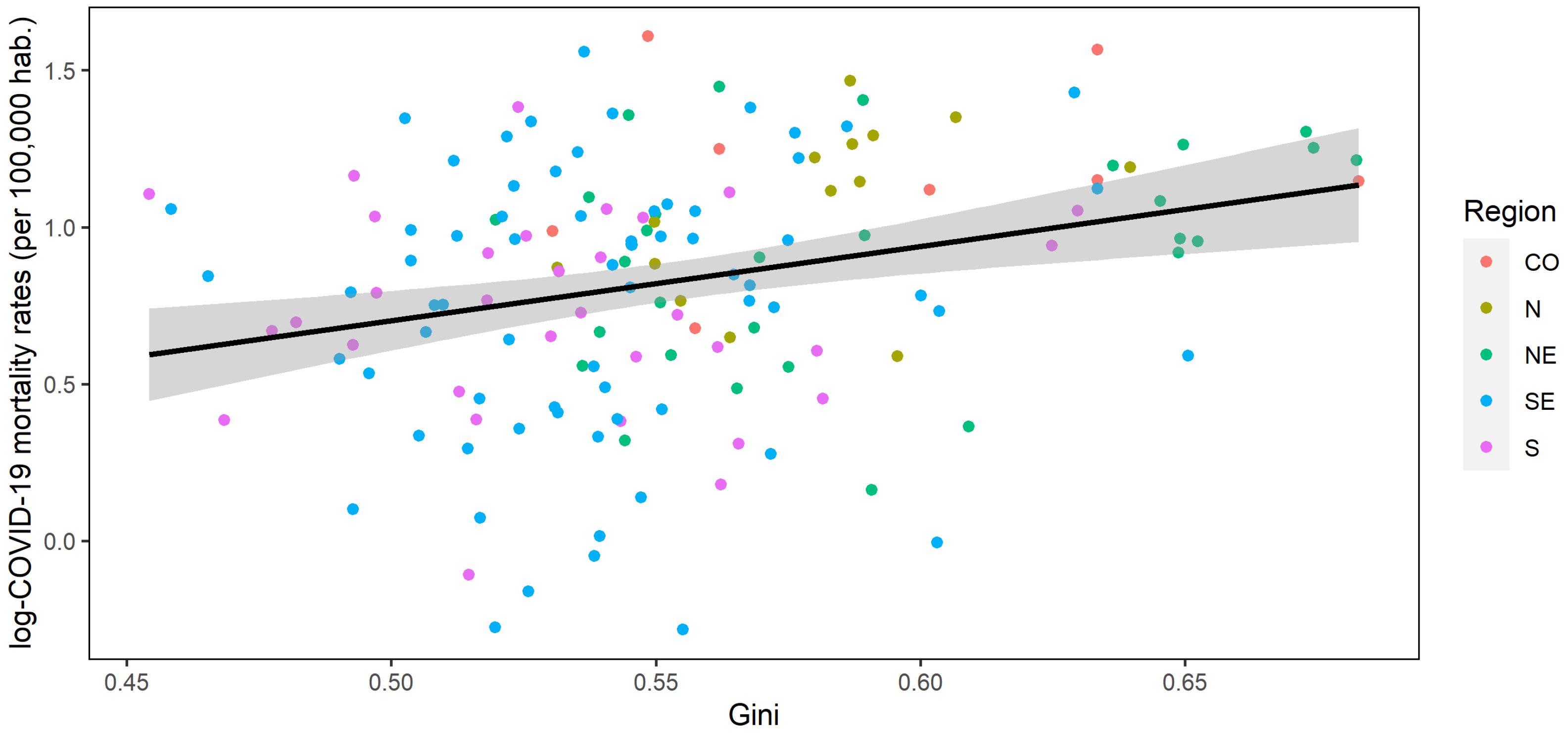

S7 Fig. Scatterplots of the logarithm of COVID-19 mortality rates (per 100,000 hab., aggregated across all weeks) versus the Gini index for each city.

The solid black line represents the LOESS fit, with its 95% confidence interval given in the shaded bands. CO = *Centro-Oeste* (Central-West), N = *Norte* (North), NE = *Nordeste* (Northeast), S = *Sul* (South), SE = *Sudeste* (Southeast).
