# Supplementary material for: Association of urban inequality and income segregation with COVID-19 mortality in Brazil
Source: PLoS One. 2022 Nov 15;17(11):e0277441. doi: 10.1371/journal.pone.0277441 (PMC9665357; doi:10.1371/journal.pone.0277441)
Supplement: S1 File — (DOCX) [file pone.0277441.s010.docx]

**Supporting information to “Association of urban inequality and income segregation with COVID-19 mortality in Brazil”**

**Negative Binomial GLMM specification**

The “fully adjusted” model in the paper corresponds to

$$Y_{it}|X_{it}\sim Negative Binomial\left( \mu_{it}, \phi\right),$$

where $Y_{it}$ is the COVID-19 death count at the $i$th city and $t$th week, $i=1, \ldots, I$, $t=1, \ldots, T$ (there are a total of $I$ = 152 cities and $T$ = 41 weeks in the sample). Here, $X_{it}$ represents all the conditional information in the model (i.e. exposures, adjustments, and random effects) and $\phi\geq0$ is a dispersion parameter such that the smaller its value, the smaller the overdispersion is (the limiting case $\phi\to+\infty$ corresponds to the Poisson, i.e. equidisperse, case). Since a logarithm link was assumed, the log of the conditional mean $\mu_{it}$ satisfies

$$\log\left( \mu_{it} \right)=o_{it}+ \alpha_{0}+\alpha_{i}+ \alpha_{t}+\sum_{k=1}^{8} \gamma_{k}A_{i}^{k}+\beta E_{i},$$

where $o_{ij}=log(N_{it}/{10}^{5})$is the model offset ($N_{it}$ is the population projections in the $i$th city, which is actually time-invariant), $\alpha_{0}$ is the model intercept, $\alpha_{i}$ is a city-level random intercept, $\alpha_{t}$ is a week-level random intercept, the $A_{i}^{k}$’s are the 8 adjustment covariates (percentage of the population aged 65 years or above, GDP per capita, population educational attainment score, poverty index, number of hospital beds per capita, number of general practitioners per capita, number of nurses per capita and the proportion of the population with access to private health insurance), with corresponding slope coefficients $\gamma_{k}$ and $E_{i}$ is the model exposure (either inequality or segregation; there is one fully-adjusted model for each), with slope coefficient $\beta$. Note that the covariates $A_{i}^{k}$ and exposures $E_{i}$ are time-invariant, and that $A_{i}^{4}$ (the poverty index) had to be dropped from the models due to collinearity with segregation.

As stated in the paper, the model setting stated above allows for explicitly modelling the outcome as mortality rates, since by defining the COVID-19 mortality rate at the $i$th city and $t$th week by $R_{it}={10}^{5}\times{(Y}_{it}/N_{it})$ and using basic properties of the logarithmic function, we then have

$$\log\left( R_{it} \right)=\log\left( {10}^{5}\times\frac{Y_{it}}{N_{it}} \right)$$

$$=\log\left( Y_{it} \right)+\log\left( \frac{{10}^{5}}{N_{it}} \right)$$

$$=\log\left( Y_{it} \right)-\log\left( \frac{N_{it}}{{10}^{5}} \right)$$

$$=\log\left( Y_{it} \right)-o_{it}$$

$$\Longleftrightarrow\log\left( Y_{it} \right)=\log\left( R_{it} \right)+o_{it}.$$

Finally, the parameterization we adopt here for the Negative Binomial is

$$P\left( Y_{it}=n \right)=\frac{\Gamma\left( n+\phi\right)}{\Gamma\left( n+1 \right)\Gamma\left( \phi\right)}\left( \frac{\phi}{\phi+\mu_{it}} \right)^{\phi}\left( \frac{\mu_{it}}{\phi+\mu_{it}} \right)^{n} , n=0, 1, \ldots$$

where $\Gamma\left( x \right):=\int_{0}^{+\infty} s^{x-1}e^{-s}ds$ denotes the Gamma function. This parameterization ensures that the conditional expectation of $Y_{it}$ given $X_{it}$ is $E(Y_{it}|X_{it}) =\mu_{it}$ and that the corresponding conditional variance is $V(Y_{it}|X_{it}) =\mu_{it}+\mu_{it}^{2}/\phi$, so that the overdispersion (relative to the Poisson distribution) is equal to $\mu_{it}^{2}/\phi$.

**Log-rates clustering analyses model specification**

Our model for the logarithm of COVID-19 mortality rates is

$$\log\left( 1+R_{its} \right)= \alpha_{0}+\alpha_{i}+\alpha_{s}+ +\varepsilon_{its}, \varepsilon_{its}\sim iid N\left( 0, \sigma_{week}^{2} \right),$$

where $R_{its}={10}^{5}\times{(Y}_{its}/N_{its})$ is the COVID-19 mortality rate at the $i$th city, $t$th week and $s$th state, and $Y_{its}$ and $N_{its}$ are the corresponding death counts and population at risk. We add “1” to each rate prior to log-transforming to ensure that the log-rates are well-defined when the rates are very small or equal to zero. Here we also assume that $\alpha_{i}\sim N\left( 0, \sigma_{city}^{2} \right)$, $\alpha_{s}\sim N\left( 0, \sigma_{state}^{2} \right)$ and that $\alpha_{i}$, $\alpha_{s} a$nd $\varepsilon_{its}$ are all independent of each other for all $i$, $t$ and $s$.

In this setting, the total model variance is given by

$$\sigma_{total}^{2}:= \sigma_{week}^{2}+\sigma_{city}^{2}+ \sigma_{state}^{2},$$

and therefore, the percentage of the variance attributed to each level is, respectively,

$$\sigma_{week}^{2}\left( \% \right):=\frac{\sigma_{week}^{2}}{\sigma_{total}^{2}}\times100\%, \sigma_{city}^{2}\left( \% \right):=\frac{\sigma_{city}^{2}}{\sigma_{total}^{2}}\times100\%, \sigma_{state}^{2}\left( \% \right):=\frac{\sigma_{state}^{2}}{\sigma_{total}^{2}}\times100\%.$$

**Sensitivity analysis: results obtained without “balancing” the sample**

Figure S1 contains a forest plot of the estimated associations between COVID-19 mortality rates and inequality or segregation across all estimated models, without “balancing” the sample (i.e. ensuring that all cities have the same number of weeks in the sample) beforehand. Although point estimates and confidence intervals are numerically different, there are no qualitative changes to the results presented in the main text.

**Additional methods and results**

Table S1 contains the definitions and sources for each variable used in the paper, S1 Fig. is the correlation matrix between all exposures and the covariates, S2 Fig. contains the Directed Acyclic Graph (DAG) of the causal associations and relationships between income inequality and income segregation with COVID-19 mortality hypothesized for this study. S3 Fig. contains the correlation matrix between all the covariates and exposures used in the main (multilevel negative binomial regression) analyses, and S4 and S5 Figs. contain the Variance Inflation Factors (VIFs) for the fully adjusted models with Gini and segregation as the exposures, respectively. S6 Fig. shows the spatial distribution of city-level COVID-19 mortality rates aggregated over four different periods. Finally, S7 and S8 Figs. contain the scatterplots between the logarithm of COVID-19 mortality rates (averaged over all weeks for each city) and Gini and segregation across cities.

**References**

1. Cota W. Monitoring the number of COVID-19 cases and deaths in Brazil at municipal federative units level. *Scielo Prepr*. Published online 2020.

2. Brasil. Ministério da Saúde. 2021. Sistema de Informação sobre Mortalidade - SIM.

3. Brasil. Ministério da Saúde. 2021. Sistema de Informação de Vigilância Epidemiológica da Gripe - SIVEP Gripe.

4. Haughton J ;Shahidur R, Khandker. *Handbook on Poverty and Inequality*. The International Bank for Reconstruction and Development/The World Bank; 2009.

5. Santos MI dos, Santos GF dos, Freitas A, et al. Urban income segregation and homicides: An analysis using Brazilian cities selected by the Salurbal project. *SSM - Popul Heal*. 2021;14(April). doi:10.1016/j.ssmph.2021.100819

6. Bilal U, Hessel P, Perez-Ferrer C, et al. Life expectancy and mortality profiles are highly heterogeneous in 363 cities of Latin America: the SALURBAL project. *Nat Med*. 2021;27(March 2021):463-470.

7. IBGE. Produto Interno Bruto dos Municípios. Published online 2010. *Inst Bras Geogr e Estatística 2010*.

8. Ortigoza AF, Tapia Granados JA, Miranda JJ, et al. Characterising variability and predictors of infant mortality in urban settings: findings from 286 Latin American cities. *J Epidemiol Community Health*. 2021;75(3):264-270. doi:10.1136/jech-2020-215137

9. IBGE. Censo Demográfico Brasileiro 2010. *Inst Bras Geogr e Estatística 2010*.
